# Effects of passenger terminal in Mashhad on citizen safety and urban traffic

**Published:** 2019-08

**Authors:** Mohammad Ebrahim Amin Ghafoori, Bahram Farhadinia, Saeed Rezaee

**Affiliations:** ^*a*^ Mashhad Municipality, Quchan University of technology, Quchan, Iran.; ^*b*^ Quchan University of Technology, Quchan, Iran.

## Abstract

**Background:**

Urban development and changes have been made in Mashhad. An outlying urban passenger terminal creates many safety and traffic problems for citizens. The placement of an intercity bus terminal along with it has increased environmental pollution, safety and traffic. Their only access is Imam Reza Street. On the other hand, this street provides a large linkage of urban roads with the great Asian route. And there are a lot of demands for it. Which interferes with the location of these terminals. In this paper, we simulate the simulation of these terminals with software, and by analyzing it, the scenario of the interchange of bus interchange terminal, modeling the change of access to an outlying urban terminal, and examining the results. Targets: Identify the status quo Identify insecure points Implement different strategies to improve the status quo.

**Methods:**

Data collection and analysis with software SPS and easy fit Use Google Earth Maps for modeling Simulation with software Anylogic.

**Results:**

Inappropriate design of the terminal entrance and exit doors The Effects of Intercity Urban Buses with Urban Traffic Unsuitable buses stop at Imam Reza Street for riding and disembarking passengers Change the location of the bus terminal entrance Create a safe station for buses.

**Conclusions:**

Given the existing damage, it is recommended. To reduce environmental pollution, reduce the safety of citizens, and reduce the traffic flow of the entrance and exit doors of an outlying urban passenger terminal to the Asian highway by providing independent access. And a secure station for it.

**Keywords:**

Traffic, Safety, Terminal

